# Synthesis of thiophene derivatives and their anti-microbial, antioxidant, anticorrosion and anticancer activity

**DOI:** 10.1186/s13065-019-0569-8

**Published:** 2019-04-18

**Authors:** Rashmi Shah, Prabhakar Kumar Verma

**Affiliations:** Department of Pharmaceutical Sciences, Maharshi Dayanand University, Rohtak, Haryana 124001 India

**Keywords:** Antimicrobial, Antioxidant, Anticorrosion, Anticancer

## Abstract

**Background:**

A new series of thiophene analogues was synthesized and checked for their in vitro antibacterial, antifungal, antioxidant, anticorrosion and anticancer activities.

**Results:**

A series of ethyl-2-(substituted benzylideneamino)-4,5,6,7-tetrahydrobenzo[*b*]thiophene-3-carboxylate derivatives were synthesized by using Gewald synthesis and their structures were confirmed by FTIR, MS and ^1^H-NMR. The synthesized compounds were further evaluated for their in vitro biological potentials i.e. antimicrobial activity against selected microbial species using tube dilution method, antiproliferative activity against human lung cancer cell line (A-549) by sulforhodamine B assay, antioxidant activity by using DPPH method and anticorrosion activity by gravimetric method.

**Conclusion:**

Antimicrobial screening results showed that compound **S**_**1**_ was the most potent antibacterial agent against *Staphylococcus aureus, Bacillus subtilis*, *Escherichia coli* and *Salmonella typhi* having MIC value 0.81 µM/ml and compound **S**_**4**_ also displayed excellent antifungal activity against both *Candida albicans* and *Aspergillus niger* (MIC = 0.91 µM/ml) when compared with cefadroxil (antibacterial) and fluconazole (antifungal) as standard drug. The antioxidant screening results indicated that compound **S**_**4**_ and **S**_**6**_ exhibited excellent antioxidant activity with IC_50_ values **48.45** and **45.33** respectively when compared with the ascorbic acid as standard drug. Anticorrosion screening results indicated that compound **S**_**7**_ showed more anticorrosion efficiency (97.90%) with low corrosion rate. Results of anticancer screening indicated that compound **S**_**8**_ showed effective cytotoxic activity against human lung cancer cell line (A-549) at dose of 10^−4^ M when compared with adriamycin as standard.

## Background

Heterocyclic compound are extensively distributed in nature and have versatile synthetic applicability and biological activity which implement the new approaches for the medicinal chemist to plan and organize towards the discovery of novel drugs [1]. Thiophene and its derivatives showed extensive significance in pharmaceutical field because of its varied biological and clinical applications [2].

Serious life-threatening infections have been increased because of the increase in the resistance of microorganism agents which is mainly caused by multi-drug resistant of Gram-positive and Gram-negative pathogenic bacteria [3]. So, there is an pressing need to designed an effective, potent and novel antimicrobial agents with better pharmacodynamic and pharmacokinetic properties [4]. Moreover, cancer also remains one of the primary causes of death in the world therefore effective treatments with novel medicines having improved tumor selectivity, efficacy, and safety remains desirable [5]. Ideal anticancer drugs eliminate the cancer cells without harming normal tissues [6]. Unfortunately, no currently available anticancer agents with desired therapeutic index that earned this criterion and clinical use of drugs which involves a weighing of benefits beside toxicity [7].

Recently, thiophene and its derivatives attracted researchers in expanding their potential in the field of antioxidant [8]. The main purpose of antioxidant is to neutralize free radicals in order to prevent various oxidative diseases like autoimmune, cardiovascular, and neurovascular diseases [9]. The setback of drug resistance and generation of free radicals has promoted the researchers to study and explore for new compounds to combat the problems of infection and the associated risks from generation of free radicals. Corrosion inhibition mechanism is a crucial problem which causes destruction of material (usually metal) by chemical and/or electrochemical reaction with its environment. Organic inhibitors generally containing heteroatom’s such as sulphur is found to have higher basicity and electron donating ability which act by adsorption on the metal surface and slab the active surface sites which leads in the reduction of corrosion rate [10, 11].

Thiophene derivatives show remarkable applications in different disciplines. In medicine, thiophene derivatives show antimicrobial [12], analgesic and anti-inflammatory [13], antihypertensive [14], and antitumor activity [15] while they are also used as inhibitors of corrosion of metals [16] or in the fabrication of light-emitting diodes in material science [17]. In light of afore-mentioned facts and in continuance of our research on the improvement of thiophene derivatives, we hereby report the synthesis and biological screening (i.e. antimicrobial, antioxidant, anticorrosion and anticancer) of thiophene derivatives with a variety of acceptor and donor groups.

## Results and discussion

### Chemistry

**Scheme 1:**
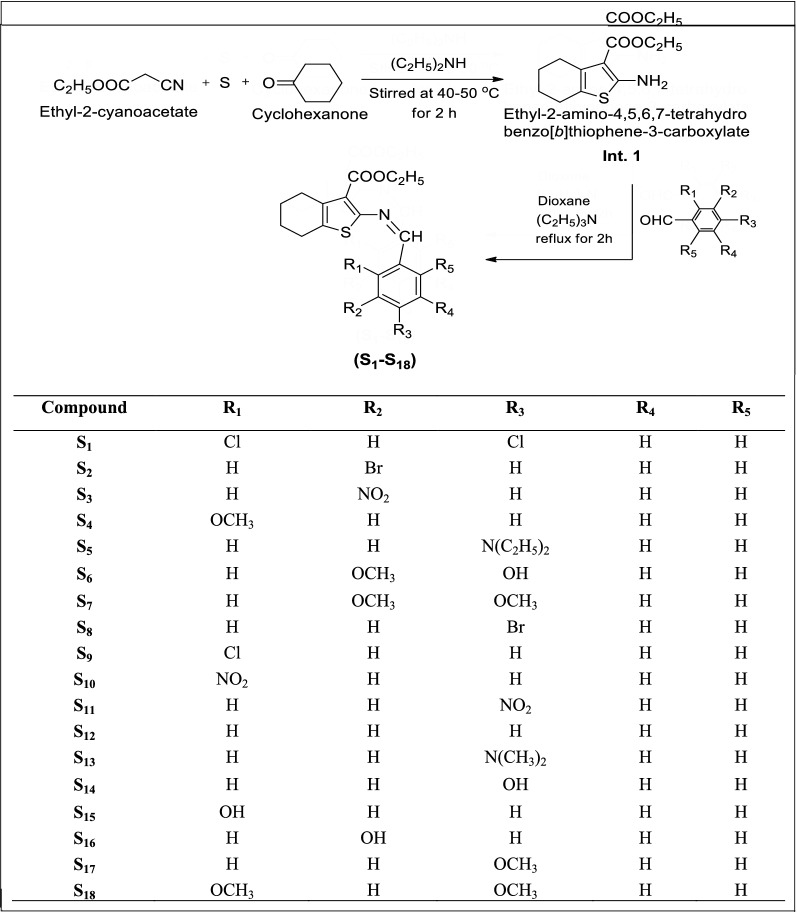
Synthesis of ethyl-2-(substituted benzylideneamino)-4,5,6,7-tetra hydrobenzo[*b*]thiophene-3-carboxylate derivatives

A series of ethyl-2-(substituted benzylideneamino)-4,5,6,7-tetrahydrobenzo[*b*]thiophene-3-carboxylate derivatives were synthesized (Scheme 1, **S**_**1**_–**S**_**18**_) by using Gewald synthesis. Initially, ethylcyanoacetate and cyclohexanone was reacted with sulphur at room temperature with continuous stirring in presence of diethylamine which resulted in the formation of intermediate-1. Later on intermediate-1 treated with substituted aromatic benzaldehyde and dioxane in presence of triethylamine yielded the final compounds (**S**_**1**_–**S**_**18**_). The structures of the entire newly synthesized compound were checked by IR data and ^1^H NMR which was in full favour with molecular structures assigned. Its physical characterizations were indicated in Table 1.

**Table 1:** The physicochemical properties of newly synthesized derivatives (S_1_–S_18_)

Compound	M. formula	M. Wt.	m. p. (°C)	R_f_ value^a^	% Yield
**S**_**1**_	C_18_H_17_Cl_2_NO_2_S	382.30	96–97	0.53	84.71
**S**_**2**_	C_18_H_18_BrNO_2_S	392.31	122–124	0.47	75.00
**S**_**3**_	C_18_H_18_N_2_O_4_S	358.41	87–89	0.62	82.22
**S**_**4**_	C_19_H_21_NO_3_S	343.44	95–98	0.38	72.67
**S**_**5**_	C_22_H_28_N_2_O_2_S	384.53	98–100	0.39	78.86
**S**_**6**_	C_19_H_21_NO_4_S	359.44	95–97	0.66	82.43
**S**_**7**_	C_20_H_23_NO_4_S	373.13	97–99	0.45	83.67
**S**_**8**_	C_18_H_18_BrNO_2_S	392.31	94–95	0.42	87.62
**S**_**9**_	C_18_H_18_ClNO_2_S	347.86	87–88	0.41	85.63
**S**_**10**_	C_18_H_18_N_2_O_4_S	358.41	94–97	0.61	79.80
**S**_**11**_	C_18_H_18_N_2_O_4_S	358.41	130–131	0.58	72.58
**S**_**12**_	C_18_H_19_NO_2_S	313.41	90–93	0.31	68.72
**S**_**13**_	C_20_H_24_N_2_O_2_S	356.48	134–135	0.53	83.23
**S**_**14**_	C_18_H_19_NO_3_S	329.41	110–111	0.68	73.65
**S**_**15**_	C_18_H_19_NO_3_S	329.41	108–109	0.63	81.35
**S**_**16**_	C_18_H_19_NO_3_S	329.41	93–94	0.56	69.87
**S**_**17**_	C_18_H_19_NO_3_S	343.44	93–95	0.55	81.81
**S**_**18**_	C_20_H_23_NO_4_S	373.13	97–99	0.62	76.69

^a^TLC mobile phase-benzene

### Antimicrobial activity

In vitro antimicrobial activity of novel synthesized compounds were screened against the microorganisms such as Gram positive (*Staphylococcus aureus, Bacillus subtilis*), Gram negative (*Escherichia coli, Salmonella typhi*) and fungal strains (*Aspergillus niger* and *Candida albicans*) using tube dilution method. The results after examined were expressed as minimum inhibitory concentration (MIC i.e. lowest conc. required for the test substances to complete growth inhibition). The MIC values of standard and synthesized compounds were presented in Table 2. Antimicrobial evaluation results indicated that the entire synthesized compounds were found to have significant antimicrobial activity and different compound were found to be active against different microorganisms.

**Table 2:** Antimicrobial activity of newly synthesized compounds (S_1_–S_18_)

Compound	Minimum inhibitory concentration (MIC = µM/ml)					
	Bacterial species				Fungal species	
	*S. aureus*	*B. subtilis*	*E. coli*	*S. typhi*	*A. niger*	*C. albicans*
**S**_**1**_	*0.81*	*0.81*	*0.81*	*0.81*	3.26	3.26
S_2_	3.19	3.19	1.59	1.59	3.19	3.19
S_3_	3.49	3.49	3.49	3.49	3.49	3.49
**S**_**4**_	3.64	3.64	3.64	3.64	*0.91*	*0.91*
S_5_	3.25	3.25	3.25	3.25	3.25	3.25
S_6_	3.48	3.48	3.48	3.48	1.74	1.74
S_7_	3.35	3.35	6.70	6.70	3.35	3.35
S_8_	3.19	3.19	3.19	3.19	3.19	3.19
S_9_	3.59	3.59	3.59	3.59	3.59	3.59
S_10_	3.49	3.49	3.49	3.49	3.49	3.49
S_11_	0.87	1.74	3.49	3.49	3.49	6.98
S_12_	3.99	3.99	3.99	3.99	3.99	3.99
S_13_	3.51	3.51	3.51	3.51	3.51	3.51
S_14_	3.79	3.79	3.79	3.79	3.79	3.79
S_15_	3.79	3.79	7.59	7.59	7.59	3.79
S_16_	3.79	3.79	3.79	3.79	3.79	3.79
S_17_	3.64	7.28	3.64	3.64	3.64	3.64
S_18_	6.70	3.35	3.35	3.35	3.35	3.35
**Cefadroxil**	1.64	3.28	1.64	1.64		
**Fluconazole**					2.04	2.04

*Obs*. minimum inhibitory concentration in each column is showed in italics

Antimicrobial screening results showed that compound **S**_**1**_ was the most potent antibacterial agent against *B. subtilis*, *S. aureus, E. coli* and *S. typhi* having MIC value 0.81 µM/ml. Further having significant antibacterial activity, the synthesized compound **S**_**4**_ also displayed excellent antifungal activity against both *A. niger* and *C. albicans* and (MIC = 0.91 µM/ml). On the whole, antimicrobial activity results (Table 2) indicated that compounds **S**_**1**_ and **S**_**4**_ were found to be most potent antimicrobial agents.

### Antioxidant activity

**Fig. 1:**
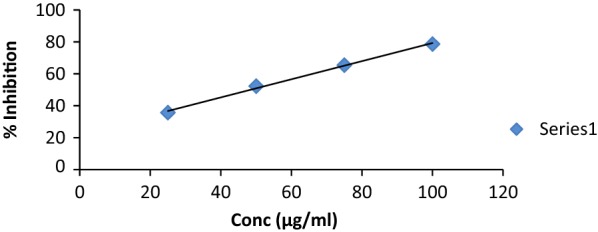
Standard graph of ascorbic acid

**Fig. 2:**
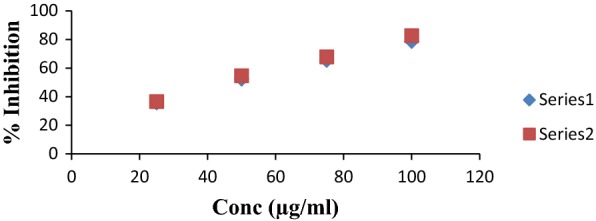
Graph of potent antioxidant compounds **S**_**4**_ and **S**_**6**_. Where Series 1 = Percentage inhibition of compound 4 and Series 2 = % inhibition of compound 6

**Fig. 3:**
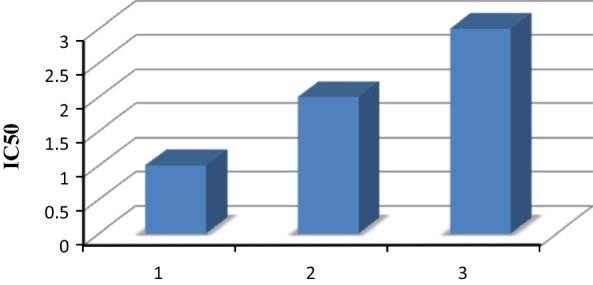
IC_50_ values of compounds **S**_**4**_ and **S**_**6**_ compared to ascorbic acid. Where 1, 2, 3 represent IC_50_ values of ascorbic acid, compounds **S**_**4**_ and **S**_**6**_ respectively

Newly synthesized derivatives were investigated for their in vitro antioxidant activity by DPPH assay at absorbance 517. The percentage (%) inhibition was calculated from the Eq. 1 and IC_50_ value of newly synthesized compounds were obtained from the graph drawn between concentrations with % inhibition of test compound given in Figs. 1, 2 and 3. From the results obtained it was known that all the newly derived compounds were found to have good to moderate antioxidant activity. Among them, compound **S**_**4**_ and **S**_**6**_ exhibited excellent antioxidant activity with IC_50_ values **48.45** and **45.33** respectively when compared with the ascorbic acid as standard drug. The results were shown in Table 3.

**Table 3:** Antioxidant activity of newly synthesized derivatives (S_1_–S_18_)

Compound	% Inhibition				IC_50_
	25 µg/ml	50 µg/ml	75 µg/ml	100 µg/ml	
S_1_	25.62	30.56	51.34	67.28	73.31
S_2_	32.47	47.61	64.92	78.52	53.12
S_3_	23.26	47.52	59.68	72.69	61.35
**S**_**4**_	35.68	52.23	65.38	78.63	*48.45*
S_5_	23.02	39.65	56.32	70.23	66.77
**S**_**6**_	36.62	54.63	67.82	82.63	*45.33*
S_7_	33.21	48.62	64.65	81.62	51.61
S_8_	15.63	32.63	44.65	66.65	73.66
S_9_	15.62	25.63	46.69	59.63	79.77
S_10_	16.53	27.58	47.25	72.35	59.01
S_11_	21.03	33.65	48.56	75.62	69.96
S_12_	15.71	32.56	44.62	65.54	76.17
S_13_	21.25	35.62	56.28	69.58	69.07
S_14_	21.68	38.91	56.74	70.73	67.11
S_15_	25.32	30.56	52.36	67.23	72.90
S_16_	21.72	36.39	58.65	72.36	66.47
S_17_	31.58	47.54	63.25	78.65	54.19
S_18_	17.63	23.56	45.63	75.63	53.05
**Ascorbic acid**	37.62	56.63	67.82	85.63	*43.35*

*Obs*. minimum inhibition concentration at 50% (IC_50_) in last column is showed in italics

### Anticorrosion activity

Corrosion inhibition of most of the active synthesized thiophene derivatives was evaluated by weight loss of mild steel in their presence and absence in 1.0 M HCl at 35 °C. The anticorrosion activity results of selected compound are indicated in Table 4.

**Table 4:** Corrosion inhibition efficiency of thiophene derivatives at 35 °C, for 12 h

Inhibitors	Conc. Ppm	Weight before	Weight after	Weight loss	Corrosion rate (mg/cm^2^ h)	Inhibition efficiency (%)	Surface area coverage
	Blank	0.7296	0.6706	0.059	18.27	–	–
S_1_	50	0.7187	0.7035	0.0152	4.71	74.24	0.74
	100	0.7106	0.6998	0.0108	3.34	81.69	0.82
	200	0.7185	0.7097	0.0088	2.72	85.08	0.85
	300	0.7189	0.7123	0.0066	2.04	88.81	0.89
S_2_	50	0.7292	0.7165	0.0127	3.93	76.04	0.76
	100	0.7354	0.7251	0.0103	3.19	80.57	0.81
	200	0.7055	0.6995	0.006	1.86	88.68	0.89
	300	0.7123	0.7080	0.0043	1.33	91.89	0.92
S_3_	50	0.7073	0.695	0.0123	3.81	77.68	0.78
	100	0.701	0.6911	0.0099	3.06	82.03	0.82
	200	0.7045	0.6973	0.0072	2.23	86.93	0.87
	300	0.7046	0.6990	0.0056	1.73	89.84	0.90
S_4_	50	0.7144	0.7057	0.0087	2.69	86.41	0.86
	100	0.6956	0.6888	0.0068	2.11	89.38	0.89
	200	0.7098	0.7023	0.0075	2.32	88.28	0.88
	300	0.7198	0.7123	0.0075	2.32	88.28	0.88
S_5_	50	0.7062	0.6933	0.0129	3.99	80.45	0.80
	100	0.7028	0.6941	0.0087	2.69	86.82	0.87
	200	0.7004	0.6964	0.004	1.24	93.94	0.94
	300	0.7045	0.7008	0.0037	1.15	94.39	0.94
S_6_	50	0.7057	0.6932	0.0125	3.87	77.27	0.77
	100	0.7967	0.7896	0.0071	2.20	87.09	0.87
	200	0.7177	0.7128	0.0049	1.52	91.09	0.91
	300	0.7123	0.7080	0.0043	1.33	92.18	0.92
S_7_	50	0.7208	0.7098	0.011	3.41	83.53	0.84
	100	0.7091	0.703	0.0061	1.89	90.87	0.91
	200	0.7102	0.7079	0.0023	0.71	*96.56*	0.97
	300	0.7103	0.7089	0.0014	0.43	*97.90*	0.98
S_8_	50	0.7152	0.7023	0.0129	3.99	79.84	0.80
	100	0.7333	0.7264	0.0069	2.14	89.22	0.89
	200	0.7225	0.7187	0.0038	1.18	94.06	0.94
	300	0.7046	0.7009	0.0037	1.15	94.22	0.94
S_9_	50	0.7272	0.7114	0.0158	4.89	77.10	0.77
	100	0.7221	0.712	0.0101	3.13	85.36	0.85
	200	0.7193	0.7115	0.0078	2.41	88.70	0.89
	300	0.7330	0.7260	0.007	2.17	89.86	0.90
S_10_	50	0.718	0.7047	0.0133	4.12	77.46	0.77
	100	0.7382	0.7311	0.0071	2.20	87.97	0.88
	200	0.6981	0.6934	0.0047	1.46	92.03	0.92
	300	0.712	0.708	0.004	1.24	93.22	0.93

*Obs*. the highest inhibition efficiency (%) in 7th column is showed in italics

**Fig. 4:**
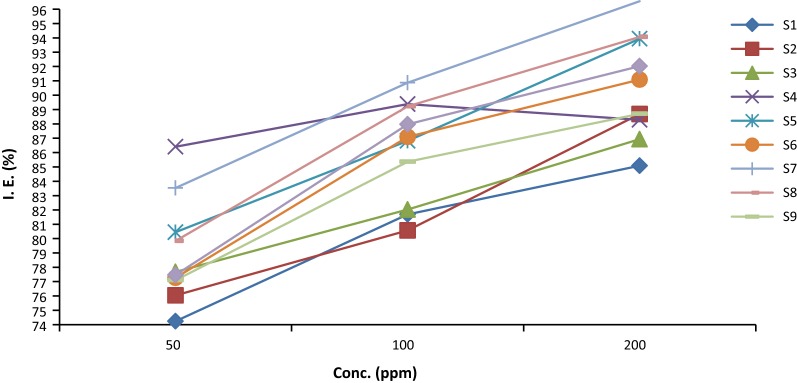
Graph of inhibition efficiency vs conc. of inhibitors for 12 h

The graph of inhibition efficiency Vs concentration at 35 °C for 12 h presented in Fig. 4. The result of inhibition efficiency after 12 h at 35 °C indicated that compounds exhibited good corrosion inhibition potential. Compound **S**_**7**_ showed more anticorrosion efficiency (97.90%) and low corrosion rate (0.98). The anticorrosion screening results indicated that the corrosion rate decreases with increasing concentration of inhibitors.

### Anticancer activity

**Fig. 5:**
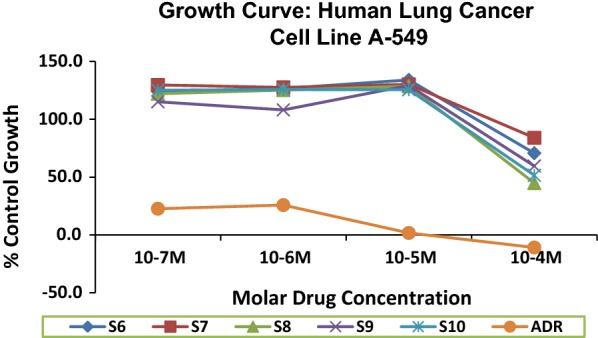
Graph plotted between tested compound and standard drug

The synthesized derivatives were screened for cytotoxicity by sulforhodamine-B (SRB) assay method on A-549 cell line and the results were compared with adriamycin as a standard drug. Among the screened compounds, compound **S**_**8**_ showed significant anticancer activity at dose 10^−4^ M against human lung cancer cell line (A-549) when compared with adriamycin as standard. Antitumor activity of the synthesized compound against human lung cancer cell line (A-549) was found to be improved because of the presence of electron withdrawing group (p-Br, Compound **S**_**8**_) on benzylidene portion. Anticancer activity of ethyl-2-(substituted benzylideneamino)-4,5,6,7-tetrahydrobenzo[*b*]thiophene-3-carboxylate derivatives were presented in Tables 5 and 6. The results obtained from graph drawn between tested compound and standard drug indicated in Fig. 5.

**Table 5:** Percentage control growth against human lung cancer cell line A-549

*1.00*	Human lung cancer cell line A-549															
	% control growth															
	Molar drug concentrations															
	Experiment 1				Experiment 2				Experiment 3				Average values			
	10^−7^ M	10^−6^ M	10^−5^ M	10^−4^ M	10^−7^ M	10^−6^ M	10^−5^ M	10^−4^ M	10^−7^ M	10^−6^ M	10^−5^ M	10^−4^ M	10^−7^ M	10^−6^ M	10^−5^ M	10^−4^ M
S6	106.9	127.4	126.8	60.4	134.1	122.0	144.3	79.4	131.3	130.6	131.0	72.2	124.1	126.6	134.0	70.7
S7	111.0	127.1	127.4	71.5	147.4	126.6	136.2	90.1	130.6	128.7	127.1	90.5	129.7	127.5	130.2	84.0
S8	101.4	131.3	126.3	33.1	134.7	115.2	127.0	45.7	130.2	129.1	132.3	55.7	122.1	125.2	128.5	44.8
S9	103.6	111.0	126.1	53.8	117.0	98.5	125.0	58.4	124.9	114.8	136.4	66.3	115.2	108.1	129.2	59.5
S10	106.8	129.6	123.0	43.1	140.2	113.2	122.9	44.9	128.4	134.1	130.6	66.7	125.1	125.6	125.5	51.6
ADR	58.0	25.8	− 0.2	− 20.2	9.3	25.4	− 1.6	− 12.8	0.7	26.3	7.1	0.3	22.6	25.8	1.8	− 10.9

**Table 6:** Anticancer activity of selected newly synthesized derivatives

A-549	Molar drug concentrations calculated from graph		
	LC_50_	TGI	GI_50_*
S_6_	NE	NE	0.048
S_7_	NE	NE	0.359
**S**_**8**_	19.5	0.126	*8E−04*
S_9_	NE	36.2	0.014
S_10_	40.84	0.222	0.001
ADR	0.199	2E−05	2E−09

*Obs*. the minimum drug concentration which causes 50% inhibition of cell growth (GI_50_) in last column is showed in italics

Where GI_50_ value of ≤ 10^−6^ molar (i.e. 1 µmolar) or ≤ 10 µg/ml is considered to demonstrate activity in case of pure compounds. For extracts, _GI50_ value ≤ 20 µg/ml is found to demonstrate activity

LC_50_: drug concentration which cause 50% cell kill; GI_50_: drug concentration which cause 50% inhibition of cell growth; TGI: drug concentration which cause total inhibition of cell growth; ADR: adriamycin, positive control compound

### Structure activity relationship (SAR) studies

The subsequent structure activity relationship of newly synthesized ethyl-2-(substituted benzylideneamino)-4,5,6,7-tetrahydrobenzo[*b*]thiophene-3-carboxylate derivatives can be derived on the basis of the result obtained from antimicrobial, antioxidant, anticorrosion and anticancer testing as follows:

1. Antibacterial activity of the synthesized compound was enhanced due to the presence of electron withdrawing group (–Cl, Compound **S**_**1**_) on benzylidene portion against *S. aureus, B. subtilis*, *E. coli* and *S. typhi.* Because of the absence of hyper conjugation of methyl group favours the increase in activity of lead molecule. The structural variations such as chloro group at o and p position to the aromatic linkage favours the activity in positive manner.
2. Antifungal activity of the synthesized compound was enhanced because of the presence of electron releasing group (o-OCH_3_, Compound **S**_**4**_) in benzylidene portion against *C. albicans* and *A. Niger.*
3. Presence of electron releasing group (o-OCH_3_, Compound **S**_**4**_, and m-OCH_3_ and p-OH, Compound **S**_**6**_) in benzylidene portion showed the significant increase in antioxidant activity.
4. Anticorrosion activity was also found to be improved because of the presence of electron releasing group (m-OCH_3_ and p-OCH_3_, Compound **S**_**7**_).
5. Anticancer activity of the synthesized compound was found to be increased against human lung cancer cell line (A-549) by the addition of electron withdrawing group (p-Br, Compound **S**_**8**_).
6. From these result we may conclude that for a compound to be most effective and potent against various target it is essential to have different structural requirements.

## Experimental section

### Materials and methods

**Fig. 6:**
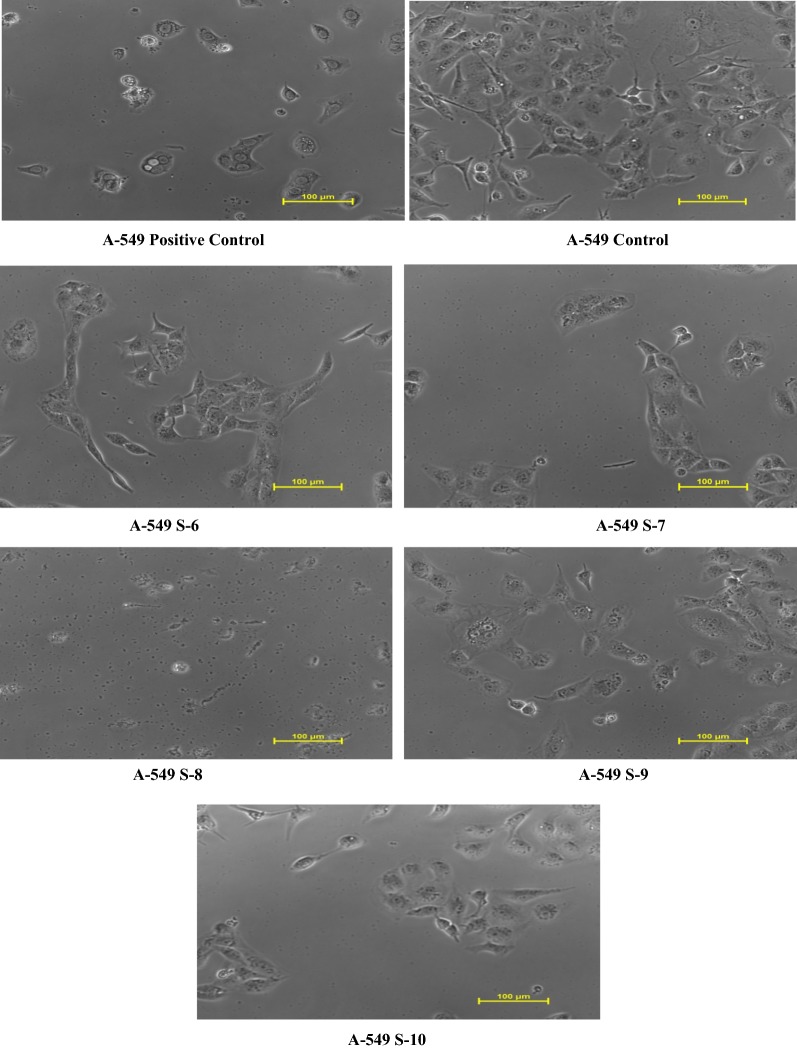
Images of in vitro testing of samples for anti-cancer activity screening

Reagents and solvents were of both laboratory and analytical grade which were used in the study. Hi-media Laboratories were used to obtain the Media for antimicrobial activity and all the microbial type cell cultures (MTCC) were obtained from Institute of Microbial Technology and Gene bank (IMTECH), Chandigarh. Labtech melting point equipment was used to determined melting points by an open glass capillary method. Thin layer chromatography (TLC) was used for observing the reaction steps forward making use of commercial silica gel plates (Merck), Silica gel F254 on aluminium sheets. ^1^H NMR spectra were screened by Bruker Avance 400 NMR spectrometer in a suitable chloroform solvent and are expressed in parts per million (δ, ppm) downfield from tetramethyl silane (internal standard). ^1^H NMR data are given as multiplicity (s, singlet; d, doublet; t, triplet; m, multiplet) and number of protons. Infrared (IR) spectra were recorded on a Bruker FTIR spectrometer using KBr pellet method and expressed in cm^−1^. The mass spectra of derived compounds were done on Waters Micromass Q-ToF Micro instrument by mass spectrometer (Fig. 6).

### General procedure for the synthesis of (ethyl-2-amino-4,5,6,7tetrahydrobenzo[*b*] thiophene-3-carboxylate derivatives (S_1_–S_18_)

#### Step I: Synthesis of ethyl-2-amino-4,5,6,7-tetrahydrobenzo[b]thiophene-3-carboxylate

Sulphur (1.92 g, 0.06 mol) was added to a mixture of ethylcyanoacetate (5.32 ml, 0.05 mol) and cyclohexanone (5.2 ml, 0.05 mol) with continuous stirring at room temperature followed by the adding up of diethylamine (5.26 ml, 0.05 mol). The reaction mixture was then stirred at 40–50 °C for 2 h. Thin layer chromatography was used for the confirmation of reaction mixture. The resulting mixture was set aside for overnight at room temperature. The precipitate obtained was filtered, washed with water, dried and recrystallized from ethanol.

#### Step II: Synthesis of ethyl-2-(substituted benzylideneamino)-4,5,6,7-tetrahydrobenzo[b] thiophene-3-carboxylate

Intermediate 1 (0.05 mol) and different substituted aromatic benzaldehyde (0.05 mol) in 15 ml of dioxane and triethylamine (0.005 mol) were taken in RBF and refluxed for 2 h. The resulting mixture was cooled and after that it was poured into crushed ice to obtain precipitate. Then was filtered and washed with 1% potassium bicarbonate followed by water, dried and recrystallized it from ethanol. The structures of synthesized derivatives were established by IR, NMR and mass analysis.

### Analytical data

**Compound S**_**1**_: Ethyl-2-(2,4-dichlorobenzylideneamino)-4,5,6,7-tetrahydrobenzo[*b*]thio-phene-3-carboxylate: M. p: 96–97 °C; yield: 84.71%; IR (KBr pellets, cm^−1^): 2977 (C–H str.), 1582 (C=C str.), 1657 (C=N str.), 1698 (C=O str., carbonyl), 1267 (C–O–C str.), 665.53 (C–S–C str., thiophene ring), 822 (C–Cl str., aromatic); ^1^H NMR (CDCl_3_, δppm): 7.28–7.50 (m, 3H, Ar–H), 8.12 (s, 1H, CH=N), 4.33 (q, 2H, CH_2_), 1.39 (t, 3H, CH_3_), 2.74 (t, 2H, CH_2_ cyclo), 1.67 (q, 2H, CH_2_ cyclo); ^13^C NMR (75 MHz, CDCl_3_) d 166.74, 163.75, 123.90, 122.84, 106.61, 51.20, 39.17, 32.08, 29.37, 27.50, 22.43; MS ES + (ToF): m/z 383 [M^+^+1].

**Compound S**_**2**_: Ethyl-2-(3-bromobenzylideneamino)-4,5,6,7-tetrahydrobenzo[*b*]thiophene-3-carboxylate: M. p: 122–124 °C; yield: 75%; IR (KBr pellets, cm^−1^): 2970 (C–H str.), 1555 (C=C str.), 1598 (C=N str.), 1707 (C=O str., carbonyl), 1269 (C–O–C str.), 682 (C–S–C str., thiophene ring), 592 (C–Br str., aromatic); ^1^H NMR (CDCl_3_, δppm): 7.28–7.78 (m, 3H, Ar–H), 9.99 (s, 1H, CH=N), 4.35 (q, 2H, CH_2_), 1.40 (t, 3H, CH_3_), 2.71 (t, 2H, CH_2_ cyclo), 1.84 (q, 2H, CH_2_ cyclo); ^13^C NMR (75 MHz, CDCl_3_) d 165.80, 161.50, 127.46, 121.60, 106.16, 51.20, 30.20, 29.39; MS ES + (ToF): m/z 393 [M^+^+1].

**Compound S**_**3**_: Ethyl-2-(3-nitrobenzylideneamino)-4,5,6,7-tetrahydrobenzo[*b*]thiophene-3-carboxylate: M. p: 87–89 °C; yield: 82.22%; IR (KBr pellets, cm^−1^): 2988 (C–H str.), 1599 (C=C str.), 1648 (C=N str.), 1686 (C=O str., carbonyl), 1272 (C–O–C str.), 697 (C–S–C str., thiophene ring), 1550, 1352 (N–O str., aromatic); ^1^H NMR (CDCl_3_, δppm): 8.04–8.93 (m, 3H, Ar–H), 10.12 (s, 1H, CH=N), 4.36 (q, 2H, CH_2_), 1.31 (t, 3H, CH_3_), 2.57 (t, 2H, CH_2_ cyclo), 1.67 (q, 2H, CH_2_ cyclo); ^13^C NMR (101 MHz, CDCl_3_) d 165.94, 160.53, 128.07, 122.37, 106.00, 50.97, 32.04, 29.75, 29.26, 29.90; MS ES + (ToF): m/z 359.41 [M^+^+1].

**Compound S**_**4**_: Ethyl-2-(2-methoxybenzylideneamino)-4,5,6,7-tetrahydrobenzo[*b*]thiophene-3-carboxylate: M. p: 95–98 °C; yield: 72.67%; IR (KBr pellets, cm^−1^): 2934 (C–H str.), 1600 (C=C str.), 1657 (C=N str.), 1693 (C=O str., carbonyl), 1252 (C–O–C str.), 647 (C–S–C str., thiophene ring), 2842 (O–CH_3_ str., aromatic); ^1^H NMR (CDCl_3_, δppm): 6.84–7.33 (m, 3H, Ar–H), 3.76 (s, 3H, CH_3_ methoxy), 9.75 (s, 1H, CH=N), 4.36 (q, 2H, CH_2_), 1.35 (t, 3H, CH_3_), 2.68 (t, 2H, CH_2_ cyclo), 1.84 (q, 2H, CH_2_ cyclo); ^13^C NMR (101 MHz, CDCl_3_) d 166.84, 161.63, 127.77, 120.37, 107.00, 50.90, 41.04, 28.75, 29.20, 28.81; MS ES + (ToF): m/z 344.4 [M^+^+1].

**Compound S**_**5**_: Ethyl-2-(4-(diethylamino)benzylideneamino)-4,5,6,7-tetrahydrobenzo[*b*] thiophene-3-carboxylate: M. p: 98–100 °C; yield: 78.86%; IR (KBr pellets, cm^−1^): 2985 (C–H str.), 1575 (C=C str.), 1649 (C=N str.), 1736 (C=O str., carbonyl), 1274 (C–O–C str.), 639 (C–S–C str., thiophene ring), 1333 (C–N str., aromatic); ^1^H NMR (CDCl_3_, δppm): 6.60–7.38 (m, 3H, Ar–H), 3.94 (q, 2H, N-CH_2_), 1.25 (t, 3H, N-CH_3_), 9.05 (s, 1H, CH=N), 4.29 (q, 2H, CH_2_), 1.34 (t, 3H, CH_3_), 2.53 (t, 2H, CH_2_ cyclo), 1.58 (q, 2H, CH_2_ cyclo); ^13^C NMR (101 MHz, CDCl_3_) d 165.50, 163.45, 129.90, 106.26, 106.82, 93.93, 74.51, 50.23, 32.24, 29.48, 22.45, 20.76, 15.11; MS ES + (ToF): m/z 385.53 [M^+^+1].

**Compound S**_**6**_: Ethyl-2-(4-hydroxy-3-methoxybenzylideneamino)-4,5,6,7-tetrahydrobenzo [*b*]thiophene-3-carboxylate: M. p: 95–97 °C; yield: 82.43%; IR (KBr pellets, cm^−1^): 2985 (C–H str.), 1597 (C=C str.), 1645 (C=N str.), 1792 (C=O str., carbonyl), 1273 (C–O–C str.), 632 (C–S–C str., thiophene ring), 3403 (O–H str., aromatic); ^1^H NMR (CDCl_3_, δppm): 7.02–7.05 (m, 3H, Ar–H), 3.76 (s, 3H, CH_3_ methoxy), 5.07 (s, 1H, OH alcohol), 9.86 (s, 1H, CH=N), 4.26 (q, 2H, CH_2_), 1.34 (t, 3H, CH_3_), 2.53 (t, 2H, CH_2_ cyclo), 1.78 (q, 2H, CH_2_ cyclo); ^13^C NMR (75 MHz, CDCl_3_) d 165.90, 164.48, 131.27, 125.37, 116.11, 54.00, 39.06, 36.17, 30.82, 39.79, 20.83, 14.26; MS ES + (ToF): m/z 362 [M^+^+1].

**Compound S**_**7**_: Ethyl-2-(3,4-dimethoxybenzylideneamino)-4,5,6,7-tetrahydrobenzo[*b*] thiophene-3-carboxylate: M. p: 97–99 °C; yield: 83.67%; IR (KBr pellets, cm^−1^): 2985 (C–H str.), 1574 (C=C str.), 1597 (C=N str.), 1648 (C=O str., carbonyl), 1274 (C–O–C str.), 639 (C–S–C str., thiophene ring), 2854 (O–CH_3_ str., aromatic); ^1^H NMR (CDCl_3_, δppm): 6.87–7.11 (m, 3H, Ar–H), 3.73 (s, 3H, CH_3_ methoxy) 9.02 (s, 1H, CH=N), 4.26 (q, 2H, CH_2_), 1.49 (t, 3H, CH_3_), 2.53 (t, 2H, CH_2_ cyclo), 1.67 (q, 2H, CH_2_ cyclo); ^13^C NMR (101 MHz, CDCl_3_) d 166.48, 163.45, 129.97, 107.25, 105.80, 94.93, 75.51, 55.23, 36.24, 29.48, 22.35, 19.76, 14.11; MS ES + (ToF): m/z 374 [M^+^+1].

**Compound S**_**8**_: Ethyl-2-(4-bromobenzylideneamino)-4,5,6,7-tetrahydrobenzo[*b*]thiophene-3-carboxylate: M. p: 94–95 °C; Yield: 87.62%; IR (KBr pellets, cm^−1^): 2985 (C–H str.), 1596 (C=C str.), 1646 (C=N str.), 1696 (C=O str., carbonyl), 1274 (C–O–C str.), 639 (C–S–C str., thiophene ring), 607 (C–Br str., aromatic); ^1^H NMR (CDCl_3_, δppm): 7.64–7.86 (m, 3H, Ar–H), 8.34 (s, 1H, CH=N), 4.81 (q, 2H, CH_2_), 1.52 (t, 3H, CH_3_), 2.51 (t, 2H, CH_2_ cyclo), 1.88 (q, 2H, CH_2_ cyclo); ^13^C NMR (75 MHz, CDCl_3_) d 165.80, 161.48, 127.24, 121.35, 106.08, 51.00, 32.06, 31.16, 29.95, 29.68, 29.50, 29.88, 25.83, 15.26; MS ES + (ToF): m/z 393 [M^+^+1].

**Compound S**_**9**_: Ethyl-2-(2-chlorobenzylideneamino)-4,5,6,7-tetrahydrobenzo[*b*]thiophene-3-carboxylate: M. p: 87–88 °C; yield: 85.63%; IR (KBr pellets, cm^−1^): 2977 (C–H str.), 1582 (C=C str.), 1657 (C=N str.), 1698 (C=O str., carbonyl), 1267 (C–O–C str.), 665 (C–S–C str., thiophene ring), 822 (C–Cl str., aromatic); ^1^H NMR (CDCl_3_, δppm): 7.17–7.74 (m, 3H, Ar–H), 10.09 (s, 1H, CH=N), 4.48 (q, 2H, CH_2_), 1.32 (t, 3H, CH_3_), 2.40 (t, 2H, CH_2_ cyclo), 1.69 (q, 2H, CH_2_ cyclo); ^13^C NMR (75 MHz, CDCl_3_) d 166.89, 161.47, 128.27, 122.37, 107.12, 51.01, 32.06, 31.17, 29.84, 29.80, 29.78, 29.68, 29.49, 29.08, 23.83, 14.26; MS ES + (ToF): m/z 349 [M^+^+1].

**Compound S**_**10**_: Ethyl-2-(2-nitrobenzylideneamino)-4,5,6,7-tetrahydrobenzo[*b*]thiophene-3-carboxylate: M. p: 94–97 °C; yield: 79.80%; IR (KBr pellets, cm^−1^): 2983 (C–H str.), 1592 (C=C str.), 1649 (C=N str.), 1699 (C=O str., carbonyl), 1268 (C–O–C str.), 696 (C–S–C str., thiophene ring), 1533, 1344 (N–O str., aromatic); ^1^H NMR (CDCl_3_, δppm): 7.64–8.24 (m, 3H, Ar–H), 10.27 (s, 1H, CH=N), 4.60 (q, 2H, CH_2_), 1.38 (t, 3H, CH_3_), 2.51 (t, 2H, CH_2_ cyclo), 1.63 (q, 2H, CH_2_ cyclo);); ^13^C NMR (75 MHz, CDCl_3_) d 166.90, 164.48, 131.27, 125.37, 116.11, 54.00, 34.06, 36.17, 30.82, 39.80, 20.93, 15.26; MS ES + (ToF): m/z 359 [M^+^+1].

**Compound S**_**11**_: Ethyl-2-(4-nitrobenzylideneamino)-4,5,6,7-tetrahydrobenzo[*b*]thiophene-3-carboxylate: M. p: 130–131 °C; yield: 72.58; IR (KBr pellets, cm^−1^): 2981 (C–H str.), 1577 (C=C str.), 1593 (C=N str.), 1713 (C=O str., carbonyl), 1269 (C–O–C str.), 690 (C–S–C str., thiophene ring), 1512, 1335 (N–O str., aromatic); ^1^H NMR (CDCl_3_, δppm): 7.98–8.24 (m, 3H, Ar–H), 10.15 (s, 1H, CH=N), 4.42 (q, 2H, CH_2_), 1.38 (t, 3H, CH_3_), 2.57 (t, 2H, CH_2_ cyclo), 1.64 (q, 2H, CH_2_ cyclo);); ^13^C NMR (75 MHz, CDCl_3_) d 165.83, 164.50, 131.37, 125.37, 117.11, 56.00, 39.05, 36.17, 30.82, 39.79, 21.83, 14.36; MS ES + (ToF): m/z 359 [M^+^+1].

**Compound S**_**12**_:Ethyl-2-(benzylideneamino)-4,5,6,7-tetrahydrobenzo[*b*]thiophene-3-carboxylate: M. p: 90–93 °C; yield: 68.72; IR (KBr pellets, cm^−1^): 2985 (C–H str.), 1601 (C=C str.), 1651 (C=N str.), 1736 (C=O str., carbonyl), 1272 (C–O–C str.), 741 (C–S–C str., thiophene ring); ^13^C NMR (75 MHz, CDCl_3_) d 165.97, 163.48, 130.27, 122.37, 107.11, 51.20, 33.06, 32.17, 30.82, 29.79, 29.70, 29.50, 29.88, 23.83, 15.26; MS ES + (ToF): m/z 314 [M^+^+1].

**Compound S**_**13**_: Ethyl-2-(4-(dimethylamino)benzylideneamino)-4,5,6,7-tetrahydrobenzo[*b*] thiophene-3-carboxylate: M. p: 134–135 °C; yield: 83.23%; IR (KBr pellets, cm^−1^): 2977 (C–H str.), 1582 (C=C str.), 1657 (C=N str.), 1698 (C=O str., carbonyl), 1267 (C–O–C str.), 665.53 (C–S–C str., thiophene ring), 822 (C–Cl str., aromatic); ^1^H NMR (CDCl_3_, δppm): 6.67–7.44 (m, 3H, Ar–H), 2.89 (s, 3H, N-CH_3_), 10.10 (s, 1H, CH=N), 4.74 (q, 2H, CH_2_), 1.38 (t, 3H, CH_3_), 2.64 (t, 2H, CH_2_ cyclo), 1.74 (q, 2H, CH_2_ cyclo); ^13^C NMR (75 MHz, CDCl_3_) d 166.89, 162.48, 137.27, 122.37, 106.00, 52.00, 33.06, 31.18, 30.82, 29.89, 29.78, 29.59, 29.88, 21.83, 14.36; MS ES + (ToF): m/z 358 [M^+^+1].

**Compound S**_**14**_: Ethyl-2-(4-hydroxybenzylideneamino)-4,5,6,7-tetrahydrobenzo[*b*]thiophene-3-carboxylate: M. p: 110–111 °C; Yield: 73.65%; IR (KBr pellets, cm^−1^): 2977 (C–H str.), 1582 (C=C str.), 1657 (C=N str.), 1698 (C=O str., carbonyl), 1267 (C–O–C str.), 665.53 (C–S–C str., thiophene ring), 822 (C–Cl str., aromatic); ^13^C NMR (75 MHz, CDCl_3_) d 165.98, 161.40, 127.24, 121.35, 106.08, 50.00, 32.66, 33.16, 29.82, 29.68, 29.50, 29.08, 21.83, 15.26; MS ES + (ToF): m/z 331.5 [M^+^+1].

**Compound S**_**15**_: Ethyl 2-(2-hydroxybenzylideneamino)-4,5,6,7-tetrahydrobenzo[*b*]thiophene-3-carboxylate: M. p: 108–109 °C; yield: 81.35%; IR (KBr pellets, cm^−1^): 2977 (C–H str.), 1582 (C=C str.), 1657 (C=N str.), 1698 (C=O str., carbonyl), 1267 (C–O–C str.), 665.53 (C–S–C str., thiophene ring), 822 (C–Cl str., aromatic); ^13^C NMR (75 MHz, CDCl_3_) d 165.00, 162.48, 128.24, 121.35, 106.08, 51.00, 32.06, 30.16, 29.82, 29.78, 29.50, 29.08, 23.83, 15.36; MS ES + (ToF): m/z 331.5 [M^+^+1].

**Compound S**_**16**_: Ethyl-2-(3-hydroxybenzylideneamino)-4,5,6,7-tetrahydrobenzo[*b*]thiophene -3-carboxylate: M. p: 93–94 °C; yield: 69.87%; IR (KBr pellets, cm^−1^): 2977 (C–H str.), 1582 (C=C str.), 1657 (C=N str.), 1698 (C=O str., carbonyl), 1267 (C–O–C str.), 665.53 (C–S–C str., thiophene ring), 822 (C–Cl str., aromatic); ^13^C NMR (75 MHz, CDCl_3_) d 165.89, 160.47, 127.27, 121.47, 106.12, 51.01, 32.06, 31.17, 29.84, 29.80, 29.88, 29.60, 29.49, 29.07, 22.84, 14.28; MS ES + (ToF): m/z 331.5 [M^+^+1].

**Compound S**_**17**_: Ethyl2-(4-methoxybenzylideneamino)-4,5,6,7-tetrahydrobenzo[*b*]thiophene-3-carboxylate: M. p: 93–95 °C; yield: 81.81%; IR (KBr pellets, cm^−1^): 2977 (C–H str.), 1582 (C=C str.), 1657 (C=N str.), 1698 (C=O str., carbonyl), 1267 (C–O–C str.), 665.53 (C–S–C str., thiophene ring), 822 (C–Cl str., aromatic); ^13^C NMR (75 MHz, CDCl_3_) d 166.89, 161.48, 127.20, 121.37, 106.11, 51.00, 32.06, 31.17, 29.80, 29.79, 29.68, 29.48, 29.08, 22.80, 14.29; MS ES + (ToF): m/z 344.4 [M^+^+1].

**Compound S**_**18**_: Ethyl-2-(2,4-dimethoxybenzylideneamino)-4,5,6,7-tetrahydrobenzo[*b*] thiophene-3-carboxylate: M. p: 97–99 °C; yield: 76.69%; IR (KBr pellets, cm^−1^): 2977 (C–H str.), 1582 (C=C str.), 1657 (C=N str.), 1698 (C=O str., carbonyl), 1267 (C–O–C str.), 665.53 (C–S–C str., thiophene ring), 822 (C–Cl str., aromatic); ^13^C NMR (75 MHz, CDCl_3_) d 165.70, 161.57, 127.27, 121.37, 107.12, 52.01, 33.06, 31.18, 29.83, 29.81, 29.78, 29.58, 29.49, 29.09, 23.83, 14.27; MS ES + (ToF): m/z 374 [M^+^+1].

### Evaluation of antimicrobial activity

Newly derived derivatives were screened for in vitro antimicrobial activity against Gram positive bacteria: *Bacillus subtilis* (MTCC 441), *Staphylococcus aureus* (MTCC 3160), Gram negative bacteria *Salmonella typhi* (MTCC 3216), *Escherichia coli* (MTCC 443) and fungal strains *Candida albicans* (MTCC 227) and *Aspergillus niger* (MTCC 281) by tube dilution method [18] and results were compared with fluconazole (antifungal) and cefadroxil (antibacterial) as standard drugs. The stock solutions were prepared in DMSO having 100 µg/ml concentrations for standard and test drugs. Fresh pure cultures were used to prepare the bacterial and fungal inoculums. In the test-tubes containing serial dilutions (50, 25, 12.5, 6.25 and 3.12 µg/ml) of test and standard compounds in nutrient broth and Sabouraud dextrose broth, 100 µl of inoculum was added. After that it was incubated at 37 ± 1 °C for 24 h (bacteria), at 25 ± 1 °C for 7 days (*A. niger*) and at 37 ± 1 °C for 48 h (*C. albicans*). Antimicrobial screening results were recorded in terms of lowest concentration of test substances which inhibited the growth of microorganisms i.e. MIC.

### Evaluation of antioxidant activity by DPPH (1,1-diphenyl-2-picrylhydrazyl) method

Antioxidant activity was evaluated spectrophotometrically by using free radical scavenging method i.e. DPPH assay. The DPPH is a stable free radical and it reacts with hydrogen donors showing the reduction to its corresponding hydrazine with the maximum absorption at 517 nm. Colour of DPPH changes from violet to yellow, indicating the reaction of DPPH with an antioxidant agent as it can donate hydrogen to get reduced with a considerable decrease in absorption at 517 nm. DPPH solution (3 µg/ml) was prepared in methanol. The solution of methanol and DPPH (1:1) was used for blank reference. Four dilutions of different concentrations (25 µg/ml, 50 µg/ml, 75 µg/ml, 100 µg/ml) of each synthesized compound and standard (ascorbic acid) were prepared in the methanol and 1 ml of each concentration was added to 1 ml of DPPH solution. The solution mixture was placed in dark place for 30 min at room temperature after vigorous shaking and their absorbance was measured by UV at 517 nm [19]. Percentage (%) inhibition of free radical DPPH was calculated as follows:

1 $$\% {\text{ Inhibition }} = \frac{{{\text{A}}_{\text{Blank}} {-}{\text{ A}}_{\text{Sample}} }}{{{\text{A}}_{\text{Blank}} }} \times 100$$

where, A_Blank_ = absorbance of the blank reaction, A_Sample _ = absorbance of the test compounds.

IC_50_ value was calculated from the graph plotted between % inhibition and synthesized derivatives. The antioxidant screening results indicated that few synthesized compound exhibited significant antioxidant activity while other showed good to moderate antioxidant activity. In them, compound **S**_**4**_ and **S**_**6**_ exhibited excellent antioxidant activity and found to have IC_50_ value and (%) inhibition comparable to ascorbic acid.

### Evaluation of anticorrosion activity by gravimetric method (weight loss)

The corrosion medium was made by dilution of 1.0 M HCl of analytical grade 37% with double distilled water. The test solutions were made up by dilution of 0.5 g thiophene derivatives with 250 ml of 1.0 M HCl solution to make 200 ppm solution (stock solution). The desired concentrations (50, 100, 200, 300 ppm) of thiophene derivatives were prepared from stock solution of 200 ppm.

The gravimetric method is commonly used corrosion monitoring method. The weight loss study has been carried out in 1.0 M HCl solution. Firstly, all mild steel samples were cut into 1 × 3 cm then scrapped with emery paper of different grade (100–1000) and rinsed with distilled water, acetone and after all dried between filter paper and weighed. The weight loss study was carried out in 25 ml of 1.0 M HCl solution filled in 50 ml beaker in presence and absence of various concentration of corrosion inhibitor for 12 h at 35 °C. After the immersion of sample in test solution, specimens were put outside and washed with double distilled water and acetone, dried and weighed again. This process of weight loss study was carried out in triplicate and the average weight loss was calculated. The % corrosion inhibition efficiency and surface coverage were calculated by following equations.

2 $$\eta w = \frac{Wo - W}{Wo} \times 100$$

3 $$\theta = \frac{Wo - W}{Wo}$$

where, Wo = weight loss value of mild steel in absence of test compound, W = weight loss value of mild steel in presence of test compound.

The equation used for calculation of corrosion rate of mild steel was given by:

4 $${\text{CR}}\,\left( {{\text{mmy}} - 1} \right) = \frac{{87.6 \times {\text{W}}}}{\text{AtD}}$$

where, W = weight loss of mild steel (mg), A = area of sample (cm^2^), t = exposure time (h) and D = density of mild steel (g cm^−3^).

### Evaluation of in vitro anticancer activity

Sulforhodamine-B (SRB) assay method was used for screening in vitro cytotoxicity of the synthesized thiophene derivative on A-549 (human lung cancer cell line) [20]. Its mechanism is focused on the capability to bind electrostatically, the protein dye sulforhodamine B and pH dependent on protein basic amino acid residues of trichloroacetic acid-fixed cells. Anticancer screening results were shown as GI_50_ (concentration of drug causing 50% inhibition of cell growth) and the results were matched with the standard anticancer drug i.e. adriamycin.

The entire synthesized compounds which have to be screened initially at dose (10^−7^ to 10^−4^) were submitted at anti-cancer screening facility (ACDSF) at ACTREC Mumbai (Tata Memorial Centre).

## Conclusion

Summarizing, we may conclude that novel thiophene derivatives were synthesized with different donor or acceptor groups on aromatic rings. Compound **S**_**1**_ showed the highest activity against various Gram positive and Gram negative bacterial strains. The di-halogen substituted derivatives were found to have significant antibacterial activities, particularly the compounds having two substituted chloro groups at the same ring. Compound **S**_**4**_ emerged as the most potent antifungal agent because of the presence of o-OCH_3_ group. Compound **S**_**4**_ and **S**_**6**_ exhibited excellent antioxidant activity and compound **S**_**7**_ showed more anticorrosion efficiency with low corrosion rate due to the presence of electron releasing groups on benzylidene portion which found to have higher basicity and electron donating ability which act by adsorption on the metal surface and block the active surface sites, thus reducing the corrosion rate. Compound **S**_**8**_ possessed significant cytotoxicity GI_50_ = 8E−04 molar against human lung cancer cell line (A-549). The results of anticancer screening indicated that the synthesized compounds having electron withdrawing groups (*p*-Br) on benzylidene portion found to possessed significant activity. So, these thiophene derivatives unquestionably grasp a greater assure in discovering as lead compound for the progress of novel therapeutic agents.

## Abbreviations

ACDSF: anti-cancer screening facility
CR: corrosion rate
DPPH: 1,1-diphenyl-2-picrylhydrazyl
HCl: hydrochloric acid
IR: infrared
IMTECH: Institute of Microbial Technology and Gene bank
MS: mass spectroscopy
MTCC: microbial type cell cultures
MIC: minimum inhibitory concentration
NMR: nuclear magnetic resonance
Ppm: parts per million
SRB: sulforhodamine-B
TLC: thin layer chromatography
